# Effects of periodontitis on micro and macro vascular complications of diabetes: A systematic review

**DOI:** 10.34172/japid.026.3353

**Published:** 2025-12-04

**Authors:** Farhan Musaie, Setareh Garousi, Mehregan Shahrokhi, Mahsa Babareshani, Danial Zamani, Zahra Ashrafi, Ala Shadbin, Ali Rezvanimehr, Zahra Sanei, Mehran Mottahedi, Nima Rahimipetrudi, Nozhan Azimi, Niloofar Deravi, Fatemeh Arabpour, Hamed Taheri

**Affiliations:** ^1^School of Dentistry, Tehran Islamic Azad University of Medical Sciences, Tehran, Iran; ^2^Faculty of Medicine, Mashhad University of Medical Sciences, Mashhad, Iran; ^3^School of Medicine, Shiraz University of Medical Sciences, Shiraz, Iran; ^4^School of Allied Medical Sciences, Tehran University of Medical Sciences, Tehran, Iran; ^5^School of Medicine, Shahid Beheshti University of Medical Sciences, Tehran, Iran; ^6^Network of Immunity in Infection, Malignancy and Autoimmunity (NIIMA), Universal Scientific Education and Research Network (USERN), Tehran, Iran; ^7^Faculty of Medicine, Mazandaran University of Medical Sciences, Sari, Iran; ^8^Cardiovascular Research Center, Shahid Mohammadi Hospital, Hormozgan University of Medical Sciences, Bandar Abbas, Iran; ^9^Dentofacial Deformities Research Center, Research Institute for Dental Sciences, Shahid Beheshti University of Medical Sciences, Tehran, Iran; ^10^Student Research Committee, School of Medicine, Shahid Beheshti University of Medical Sciences, Tehran, Iran; ^11^Department of Orthodontics, School of Dentistry, Shahid Sadoughi University of Medical Sciences, Yazd, Iran; ^12^Dental School, Kazan Federal University, Tatarstan, Russia

**Keywords:** Diabetes mellitus, Macrovascular complications, Microvascular complications, Periodontitis, Risk factors

## Abstract

**Background.:**

Periodontitis (PD) is a chronic inflammatory disease of the oral cavity. PD can adversely affect glycemic control and cause macro- and micro-vascular diseases in diabetic patients. This article aims to systematically review the association between periodontitis and micro- and macro-vascular complications of diabetes.

**Methods.:**

This review was conducted under the Preferred Reporting Items for Systematic Reviews and Meta-analysis (PRISMA). A thorough search of the Scopus and PubMed databases was performed up to 2022. English articles were included.

**Results.:**

In this study, seven studies with 29,679 participants investigated the relationship between periodontitis and micro- and macro-vascular complications. There were two cohort studies, six cross-sectional studies, and one case‒control study. Follow-up durations ranged from 6 months to 11.64 years. The reports were published between 2002 and 2022. There was a relationship between periodontitis and the microvascular complications of diabetes in four studies. One study found no significant association between periodontitis and the microvascular complications of diabetes. The correlation between coronary heart disease (CHD) and periodontal disease was confirmed by one study. Furthermore, another study found that periodontitis increased the risk of cerebral and myocardial infarctions.

**Conclusion.:**

The present study demonstrated that diabetes mellitus (DM) patients with periodontitis are at an increased risk of macro- and micro-vascular complications, particularly myocardial and cerebral infarctions.

## Introduction

 Periodontitis (PD) is a chronic inflammatory disease of the oral cavity, caused by bacteria. Similar to many other diseases, PD can cause symptoms, including swelling, bleeding, and changes in the gingival color. Additionally, it has some effects on teeth. For instance, it causes bone damage, resulting in tooth loss. Accumulation of pus between the teeth and a bad taste are two other signs. On the other hand, this infection can cause adverse systemic effects due to the penetration of bacteria and their products into the bloodstream.^1,2^

 These products stimulate the host’s immune system, which leads to the release of inflammatory mediators into the bloodstream. These mediators can cause systemic disorders, especially in people with diabetes mellitus (DM). For example, PD adversely affects glycemic control, which has a significant relation with DM.^3,4^

 DM is caused by long-term hyperglycemia and can lead to macro- and micro-vascular diseases. Diabetic nephropathy, neuropathy, and retinopathy are some examples of microvascular complications. Coronary artery disease, stroke, and peripheral arterial disease are classified as macro-vascular diseases.^5^

 It should be noted that PD is the most common chronic infection in diabetic patients.^6^ Therefore, this article aims to systematically review the association between periodontitis and micro- and macro-vascular complications of DM.

## Methods

###  Protocol and registration 

**Figure 1:**
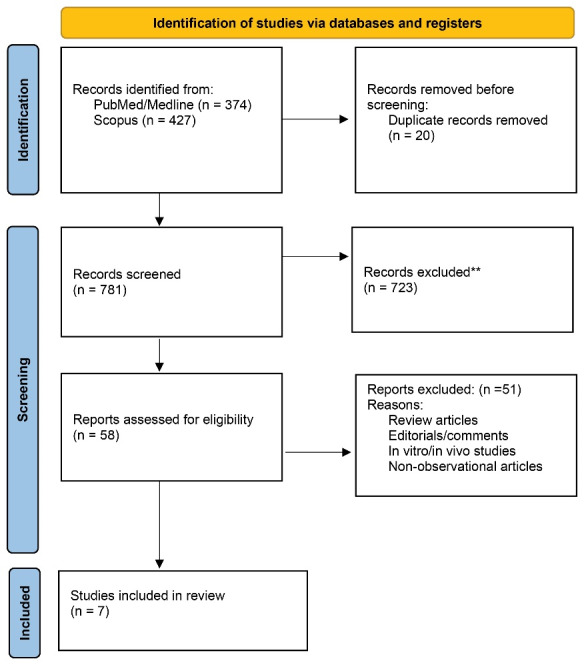
PRISMA flow diagram of the study

 PRISMA (Preferred Reporting Items for Systematic Reviews and Meta-Analyses) was followed for the present systematic review.^7^Figure 1 shows the PRISMA checklist for reporting the results of the systematic review. The study protocol was registered in Open Science Framework (https://osf.io/hncg9).

###  Search strategy

 A systematic search was conducted across the PubMed and Scopus databases (September 12, 2022) to identify relevant studies.

 The search strategy included a combination of keywords and Medical Subject Headings (MeSH) terms related to “microvascular” OR “macrovascular”, OR “diabetic ulcer”, AND “periodontitis.” The entire search strategy used for all databases is summarized in Supplementary file 1. We only considered English studies (Table S1).

###  Inclusion criteria

 Observational and clinical studies, including cross-sectional, case‒control, cohort studies, and clinical trials investigating the impact of periodontitis on micro- and macro-vascular complications of DM in humans, including diabetic retinopathy, nephropathy, neuropathy, cardiovascular disease, and death.

###  Exclusion criteria

 Systematic reviews, commentaries, editorials, in vitro studies, in vivo studies, and animal studies were excluded. Moreover, additional relevant studies were identified by hand-searching the references of articles included in the primary search.

###  Study selection 

 Six reviewers (AR, ZA, NR, DZ, MM, and SG) independently screened all the titles and abstracts after removing duplicate records. Discrepancies were resolved by consensus or using a third reviewer (AS). When studies met the inclusion criteria, the full texts were obtained and independently assessed by two authors (FM and ZS). When there was disagreement between reviewers, a third author (MB) was consulted. Finally, 7 studies were included, and studies that did not meet the inclusion criteria were excluded. No restrictions for age, gender, or race were applied.

 The screening process is shown by the 2020 PRISMA checklist in Figure 1.

###  Quality assessment 

 The quality and risk of bias of all studies that met the inclusion criteria were independently evaluated by two reviewers using the Joanna Briggs Institute (JBI) Critical Appraisal tools (https://jbi.global/critical-appraisal-tools).

 Evaluation of methods or reporting for all types of studies was carried out using this tool. For qualitative studies, the JBI tool includes 10 questions, each with four possible answers: Yes, No, Uncertain, and Not Applicable. A study was considered low risk of bias if 70% of its questions were answered “yes.” Those who responded “yes” to 50‒69% of the questions had a moderate risk of bias, while those who responded “yes” to < 50% of the questions had a high risk of bias. Studies with “good” or “fair” quality were included, and “low-quality” studies were excluded. Disagreements were resolved by consensus.

###  Data extraction 

 Five reviewers independently extracted data from articles using a pre-prepared standardized template. Authors extracted 1) authors, 2) country, 3) type of study, 4) follow-up duration, 5) population, 6) sex, 7) adjustments, 8) outcomes, and 9) quality score.

 If there was disagreement between reviewers, a third author was consulted.

## Results

 This study included 7 studies with 29,679 participants that investigated the relationship between periodontitis and micro- and macro-vascular complications in diabetic patients. These studies were conducted in Korea,^8,9^ Denmark,^10^ Thailand,^11^ Mexico,^12^ Japan,^13^ and India.^5^ Regarding study design, there were two cohort studies,^8,9^ four cross-sectional studies,^5,11-13^ and one case‒control^10^ study. The follow-up duration for cohort studies ranged from 6 months^10^ to 11.64 years.^9^ All of these reports were published between 2002 and 2022.

 Individuals with diabetes may suffer from micro-vascular complications such as nephropathy, neuropathy, and retinopathy. According to Park et al,^8^ periodontitis is an independent risk factor for diabetes-related micro-vascular diseases. Veena et al’s^5^ study revealed a significant correlation between DM and diabetic retinopathy (DR), DM duration and severity of PD, DR severity and severity of PD, PD severity and levels of higher glycated hemoglobin (HbA1c) and serum creatinine, and also a positive correlation between DR severity and levels of HbA1c and serum creatinine. Menchaca-Díaz et al^12^ indicated that periodontitis and edentulism are associated with the occurrence of neuropathy in diabetic patients. According to Nitta et al,^13^ a higher prevalence of diabetic micro-vascular complications is associated with more severe periodontitis in type 2 diabetes. In contrast, in the Rawdaree et al’s^11^ study population, DM micro-vascular complications were not significantly associated with periodontitis (Table 1).^11^

**Table 1:** A summary of the studies on the effects of periodontitis on micro- and macro-vascular complications of diabetes

**Author**	**Country**	**Type of study**	**Follow-up duration**	**Population**	**Sex (female %)**	**Adjustments**	**Outcomes**	**Quality score**
Song et al^9^	South Korea	Cohort	11.64 years	17,009 patients with DM who had participated in a nationwide health-screening program, including oral health examination, during 2002–2003 in South Korea.	35.25%	Age, gender, household income, BMI, smoking status, alcohol consumption, physical activity, presence of hypertension, levels of fasting glucose, total cholesterol	1-Periodontitis was an independent risk factor for the development of cerebral or myocardial infarction (adjusted HR: 1.17, 95% CI: 1.02–1.34; *P* = 0.030) 2- In the subgroup analysis for individual outcomes, periodontitis was an independent risk factor for cerebral infarction (adjusted HR: 1.20, 95% CI: 1.01–1.42; P = 0.040). In contrast, the presence of periodontitis was not significantly associated with an increased risk of myocardial infarction (adjusted HR: 1.11, 95% CI: 0.88–1.42; *P* = 0.384).	10/11
López et al^10^	Denmark	Case-control	6 months	61 patients with myocardial infarction and age between 30-50 years old	27.8 %	Smoking, diabetes, systolic blood pressure	1. The mean attachment level was positively associated with case status (OR: 3.17; 95% CI = [1.31; 7.65]), as was the mean pocket depth (OR: 8.64, 95% CI = [1.22; 61.20]) 2. The number of teeth present was not statistically significantly associated with case status (ORΩ0.93; 95% CI = [0.83; 1.04]).	8 /10
Rawdaree et al^11^	Thailand	Cross-sectional	-	-	60%	-	1. The mean ( ± SD) chemical attachment levels among patients with and without complications were 3.6 ( ± 1.1) mm and 3.3 ( ± 1.0) mm, respectively (*P* > 0.05). 2. The mean ( ± SD) probing depth among those with and without complications were 2.5 ( ± 0.6) and 2.5 ( ± 0.6), respectively.(*P* = 0.75) 3. On logistic regression analysis, periodontitis was not significantly associated with microvascular	5/8
Menchaca-Díaz et al^12^	Mexico	Cross-sectional study	-	436 patients with type 2 diabetes	68.3 %	Age, duration of diabetes, glycaemic control, smoking, and alcohol use	Neuropathy associated with severe periodontitis was observed (odds ratio [OR]: 2.7; 95%: confidence interval [IC]: 1.5-4.8). Neuropathy was associated with edentulism (OR: 4.4; 95% CI: 2.0-9.4). The multivariate analysis with logistic regression maintained the association between severe periodontitis and edentulism with neuropathy as significant (OR adjusted: 1.7; 95% CI: 1.1-2.6)	8 / 8
Nitta et al^13^	Japan	Cross-sectional	-	620 patients with type 2 diabetes	38.8%	Diabetes duration, sex, age, and HbA1c level	There was a significant association between the prevalence of periodontitis and glycaemic control (*P* < 0.01). The prevalence of periodontitis was associated with high HbA1c ( ≥ 8.0% [64 mmol/mol] and a longer duration of diabetes ( ≥ 15 years). The prevalence of periodontitis was not significantly associated with the number of microvascular complications. The incidence of severe periodontitis was significantly higher in patients with good, fair, and poor glycaemic control than in those with excellent glycaemic control (*P* < 0.05, *P* < 0.01, and *P* < 0.01, respectively). The severity of periodontitis was significantly associated with the number of microvascular complications (OR: 1.3; 95% Cl, 1.1–1.6), and HbA1c ≥ 8.0% (64 mmol/mol) (OR: 1.6; 95% Cl, 1.1–2.3). The incidence of severe periodontitis was significantly higher among patients with microvascular complications than among those without microvascular complications (*P* < 0.05)	8/8
Park et al^8^	Korea	Cohort	1 year	11,353 diabetes participants without prior microvascular complications	31%	Cox regression model analysis was adjusted for age, sex, alcohol consumption, smoking habits, body mass index, frequency of exercise, income level, comorbidities, and laboratory findings	periodontitis was significantly associated with the occurrence of microvascular complications. periodontitis was an independent risk factor for diabetes-related microvascular complications (adjusted HR:1.13; 95% CI:1.04–1.23; *P* = 0.004	7.9
Veena et al^5^	India	Cross-sectional study	_	Diabetes in 200 adult diabetics aged 30 to 65 years with varying severity of DR	28%	Did not any specific adjustment mention	6.8	1. duration of DM and the severity of DR and PD (*P* < 0.001). 2. The severity of PD was directly correlated with the severity of DR (*P* < 0.001). 3.association between the levels of HbA1c and serum creatinine and severity of DR and PD (*P* < 0.001). 4. Association between the mean PI and gingival index scores and severity of DR (*P* < 0.001).

BMI: Body Mass Index; CVD: Cardiovascular Disease; TC: Total Cholesterol; BP: Blood Pressure; CBG: Casual Blood Glucose; PSR: Periodontal Screening Record; DM: individuals with diabetes without neuropathy; DMN: individuals with cardiovascular autonomic diabetic neuropathy; HbA1c: hemoglobin A1C; PI: plaque index; GI: gingival index; HR: hazard ratio.

 Macro-vascular diseases are another group of complications that diabetic people may have. A positive association between coronary heart disease (CHD) and periodontal disease was corroborated by López et al.^10^ In addition, according to Song et al,^9^ myocardial and cerebral infarction are both independently associated with periodontitis. In these studies, adjustments were as follows: smoking,^8-10,12^ diabetes,^10^ systolic blood pressure age,^8,9,12,13^ duration of diabetes,^12,13^ glycemic control,^12^ alcohol use,^8,9,12^ sex,^8,9,13^ HbA1c level frequency of exercise,^8^ income level,^8,9^ body mass index,^8,9^ laboratory findings,^8^ comorbidity, physical activity,^9^ presence of hypertension, levels of fasting glucose,^9^ and total cholesterol.^9^

## Discussion

 Periodontitis, a highly prevalent disease, is associated with immune activation in response to the accumulation and maturation of oral bacteria on teeth, leading to the destruction of the supporting tissues of teeth. Periodontitis and DM, as two complex chronic diseases, are linked by a bidirectional relationship, with a two to three times higher risk of periodontitis in diabetic patients and poorer glycemic control in patients with periodontitis compared to healthy individuals. Several studies in the past 2‒3 decades demonstrated HbA1c and worse diabetic complications as side effects of periodontitis, which can be alleviated following periodontal therapy.^8,14,15^

 The present systematic review included 29,679 patients across 7 studies and examined the effects of periodontitis on micro- and macro-vascular complications of DM. Overall, the present study showed positive interactions between periodontitis and vascular factors of DM. The numerous studies that made up this systematic review showed that individuals with PD had a higher chance of developing diabetic complications than those with DM, who did not have the condition.

 DM can lead to long-term micro-vascular complications that affect small blood vessels. Retinopathy, nephropathy, and neuropathy are examples of these. The mean plaque index (PI) and gingival index (GI) scores were highest among patients with proliferative DR. PD severity and DM duration were positively correlated.^5^ According to research by Banthia et al,^16^ individuals with DR had worse dental hygiene than those with DM, but no DR.^16^ The result of periodontal destruction is noticeably worse in individuals with DR, and DM is related to PD.^17^ Menchaca-Díaz et al^12^ observed that neuropathy is associated with severe periodontitis.^12^ The multivariate analysis with logistic regression maintained that the association between severe periodontitis and edentulism with neuropathy was significant. Veena et al^5^ found a statistically significant association between the duration of DM and DR and periodontitis severity.^5^ Additionally, Nitta et al^13^ discovered that patients suffering from microvascular complications had considerably fewer teeth than patients without such complications.^13^

 Several mechanisms may explain the increased correlation between cardiovascular risk and indicators of poor oral hygiene. First, there are numerous risk factors for both periodontitis and cardiovascular conditions, including metabolic syndrome, smoking, alcohol use, obesity, hypertension, dyslipidemia, aging, and low socioeconomic level.^18-21^ Moreover, periodontitis can cause hyperglycemia and poor glucose regulation through enhanced local and systemic inflammatory reactions, raising the risk of cardiovascular disease.^3,22^ With a 17% elevated risk, periodontitis was a standalone risk factor for cerebral or myocardial infarction. Periodontitis was a 20% independent risk factor for cerebral infarction in the subgroup analysis for individual outcomes. However, the risk of myocardial infarction was not significantly enhanced when periodontitis was present.^9^

 Patients with poor dental hygiene have a variety of risk factors that can all lead to the development of cardiovascular problems. Alternatively, periodontitis and carious teeth may contribute to increased long-term cardiovascular risk.^23^ The onset and progression of atherosclerosis can both be directly influenced by oral infection and inflammation. Bacteremia from the oral cavity can penetrate the walls of blood vessels and enter the bloodstream.^24^ Additionally, periodontal bacteria’s virulence factors and oral inflammation can exert several detrimental effects on the cardiovascular system, such as oxidative stress, a rise in systemic inflammation, endothelial dysfunction, an increase in thrombotic factors, and immune cell reactions to cardiovascular tissues.^24,25^ According to Song et al,^9^ patients with DM had an elevated chance of developing myocardial or cerebral infarction due to both periodontitis and having more carious teeth. A greater number of carious teeth ( > 5) was substantially linked to an increased risk of cerebral infarction by 80%, but not to an increased risk of myocardial infarction, which increased by 41%, according to the study. Myocardial or cerebral infarction incidence was adversely correlated with frequent tooth brushing ( ≥ 2 times/day). Brushing teeth more than twice per day significantly reduced the risk of myocardial or cerebral infarction compared with brushing just once per day.^9^

 Poor glycemic control has been shown to increase the prevalence and severity of periodontitis in patients with type 2 diabetes due to chronic hyperglycemia-induced disorders with many synergistic adverse effects, including higher growth factor levels,^26^ stimulated inflammation,^27^ elevated oxidative stress,^28^ production of advanced end-products of glycation,^29^ neutrophil dysfunction,^30^ and protein kinase C activation.^31^ There was a statistically significant difference between the groups and a positive association between serum creatinine and HbA1c levels and the intensity of PD. With an average HbA1c of 9.058% and an average serum creatinine of 1.498 mg/dL, a statistically significant association was found between the intensity of DR, serum creatinine levels, and HbA1c.^5^ A total of 620 diabetic patients participated in the Nitta et al’s^13^ (2017) study. A total of 620 research participants had 293 patients with poor glycemic control (47.2%), 152 with fair glycemic control (24.5%), 135 with good glycemic control (21.8%), and 40 with excellent glycemic control (6.5%). Patients with good, medium, and poor glycemic control had a significantly higher incidence of severe periodontitis than individuals with excellent glycemic control.^13^

 The number of carious teeth, clinical assessments of periodontal health, and the oral health state were all part of the diagnostic parameters for periodontitis. Accurate reporting of diagnostic standards and pertinent medical data is essential for further studies. Due to the diverse ethnic origins of the individuals included in this analysis, the findings are likely to apply to people worldwide. The reported relationships between periodontitis and DM complications need to be confirmed in high-quality trials with clinical endpoints, despite challenges posed by follow-up periods, the number of patients involved, and drop-outs. To better understand the relationship between periodontitis and diabetic complications, further studies must provide uniform reporting of diagnostic criteria and pertinent medical data and include well-designed randomized clinical trials.

## Conclusion

 In DM patients, periodontitis was associated with the incidence of macro- and micro-vascular problems, especially myocardial and cerebral infarction, according to our study. It is well-established that various systemic illnesses and periodontitis are closely linked. PD is linked to cardiovascular problems and death, and enhances sensitivity to atherosclerosis or thrombosis. According to this, frequent toothbrushing and early detection of periodontal conditions may be useful interventions to successfully lower cardiovascular problems, which are a substantial cause of morbidity and mortality in the diabetic group. We advise careful, well-designed intervention trials to look into how periodontal therapy affects the progression of diabetic problems to potentially decrease micro- and macro-vascular consequences in patients with DM.

## Competing Interests

 The authors declare that they have no known competing financial interests or personal relationships that could have influenced the work reported in this paper.

## Data Availability

 All data generated or analyzed during this study are included in this published article and accompanying supplementary materials.

## Ethical Approval

 Not applicable.

## Supplementary Files


Supplementary file 1 contains Table S1.
