# Supplementary material for: Effects of periodontitis on micro and macro vascular complications of diabetes: A systematic review
Source: J Adv Periodontol Implant Dent. 2025 Dec 4;18(1):48–54. doi: 10.34172/japid.026.3353 (PMC12913210; doi:10.34172/japid.026.3353)
Supplement: Supplementary file 1 — contains Table S1. [file japid-18-48-s001.pdf]

Musaie et al, *J Adv Periodontol Implant Dent.* 2026;18(1):48-S1
doi: 10.34172/japid.026.3353
https://japid.tbzmed.ac.ir

Supplementary file 1

Table S1. The search strategy of PubMed and Scopus databases

Search engine		Additional filters
**Scopus**	**(TITLE(micro) OR TITLE(microvascular) OR TITLE(retinopathy) OR TITLE(nephropathy) OR TITLE(neuropathy) OR TITLE(ckd) OR TITLE(kidney) OR TITLE(macro) OR TITLE(macrovascular) OR TITLE(heart) OR TITLE(cardiac) OR TITLE(cardiovascular) OR TITLE(atrial) OR TITLE(coronary) OR TITLE(diabetic AND foot) OR TITLE(diabetic AND ulcer)) AND TITLE(periodontitis)**	English, September, 12,2022
PubMed	**((micro[Title]) OR (microvascular[Title]) OR (retinopathy[Title]) OR (nephropathy[Title]) OR (neuropathy[Title]) OR (ckd[Title]) OR (kidney[Title]) OR (macro[Title]) OR (macrovascular[Title]) OR (heart[Title]) OR (cardiac[Title]) OR (atrial[Title]) OR (cardiovascular[Title]) OR (coronary[Title]) OR (diabetic foot[Title]) OR (diabetic ulcer[Title])) AND (periodontitis[Title])**	English, September, 12,2022
